# Pure red cell aplasia in a patient with rheumatoid arthritis after JAK inhibitor exposure—diagnostic challenges

**DOI:** 10.1093/omcr/omag125

**Published:** 2026-07-12

**Authors:** Shashi Rajnarinesingh, Maria Georgiou, Nita Prasannan, Priya Sriskandarajah

**Affiliations:** Department of Haematology, Guy’s Hospital, London SE1 9RT, United Kingdom; Department of Haematology, Guy’s Hospital, London SE1 9RT, United Kingdom; Department of Haematology, Guy’s Hospital, London SE1 9RT, United Kingdom; Department of Haematology, Guy’s Hospital, London SE1 9RT, United Kingdom

**Keywords:** haematology, rheumatology, red cell aplasia, rheumatoid arthritis, JAK inhibitor

## Abstract

We describe a patient with long-standing rheumatoid arthritis (RA) who developed severe refractory transfusion-dependent anaemia lasting several months following treatment with a JAK1 inhibitor (Filgotinib). The patient proceeded with extensive investigations, including bone marrow assessment, and was initially diagnosed with Filgotinib-associated anaemia. However, despite stopping this agent, the anaemia persisted with fluctuating reticulocyte count resulting in multiple hospital admissions. Although the anaemia was initially attributed to Filgotinib, its persistence alongside delayed reticulocytopenia, ultimately led to a diagnosis of acquired Pure Red Cell Aplasia more than 12 months later. Complete remission was achieved with prednisolone + ciclosporin A. This case underscores the need to consider PRCA in RA patients on JAK inhibitors even with initially normal reticulocyte counts, as these agents may transiently mask underlying aplasia.

## Introduction

Pure red cell aplasia (PRCA) is a syndrome characterised by normocytic anaemia and reticulocytopenia, with profound reduction or absence of erythroid precursors in the bone marrow [1]. PRCA can be classified as congenital or acquired, with the latter further subclassified as either primary or secondary [2]. PRCA secondary to rheumatoid arthritis (RA) is a rare extra-articular manifestation of this disease with variable outcomes reported following immunosuppressive treatment [3].

We report the first case of PRCA apparently masked by JAK1-inhibitor (Filgotinib) therapy in RA, in which initial reticulocyte counts were normal, leading to diagnostic delay and highlighting the need for serial monitoring in patients receiving these agents.

## Case report

A 67-year-old female patient was referred to the haematology clinic with a two-month history of transfusion-dependent normocytic anaemia with recurrent hospital admissions and no evidence of gastrointestinal (GI) blood loss.

Medical history included seropositive RA, solitary sacroiliitis, interstitial lung disease, osteoporosis with previous spinal fractures and fibromyalgia. She previously had several disease modifying agents for her RA including Methotrexate, Sulfasalazine, Adalimumab, Etanercept and Abatacept. She had commenced Filgotinib a few weeks prior. The patient was wheelchair-bound due to debilitating joint disease.

Baseline blood counts on presentation were as follows: Hb 79 g/l, MCV 90 fl, MCH 31.8, WCC 9.7 × 10*9/l, Neutrophils 8.7 × 10*9/l, Lymphocytes 0.9 × 10*9/l, Eosinophils 0.1 × 10*9/l, Platelets 730 × 10*9/l, Reticulocyte count 60 × 10*9/l. Blood film examination demonstrated reactive features. Further investigations to rule out differential causes for the anaemia are summarised in Table 1.

**Table 1:** Summary of additional investigations.

Investigation	Results	Differential Diagnosis
Haematinics	Iron, B12 and folate normal	Nutritional deficiency
Erythropoietin	18.4 mU/l (NR 5-25 mU/l)	Low epo production due to renal disease
Renal Profile	75 μmol/l (NR 49-90 μmol/l)	Kidney Disease-related anaemia
Unconjugated Bilirubin Lactate Dehydrogenase Haptoglobin Coombs Test	19 μmol/l (NR < 21 μmol/l) 141 U/l (NR 125-220 U/l) 2.99 g/l (0.63—2.73 g/l) Negative	Haemolysis
CTD Profile	RF/CCP + ve, ANA homogenous 1/160 ENA -ve, myositis blot -ve Anti-dsDNA -ve	Connective tissue diseases including SLE and Sjögrens syndrome.
Viral Screen —HIV serology —Hepatitis B serology —Hepatitis C serology —Parvovirus DNA	Negative Negative Negative Negative	Infection-related anaemia
Paraprotein Serum Free Light chain ratio	Paraprotein not detected 1.48 (NR 0.26—1.65)	Multiple Myeloma
Imaging	PET/CT Whole Body Imaging: No evidence of thymoma or malignancy. Metabolically active polyarthropathy associated with multiple predominantly subcentimetre supra−/infra-diaphragmatic nodes demonstrating low-grade tracer uptake at most and diffusely increased marrow tracer uptake are favoured reactive in the context of rheumatoid arthritis with interstitial lung disease rather than secondary to an underlying haematological malignancy.	Thymoma or other malignancy
Immunophenotyping of B and T cells	No clonal B or T cells detected	Lymphoproliferative disorders
Flow cytometry screening for PNH	No PNH clone detected	Aplastic anaemia associated with PNH
T-cell clonality studies	- Polyclonal *IGH* and *IGK* gene rearrangements detected.—Conclusion – no clonal T cell population detected	Lymphoproliferative disorder including T-LGL
Lymphocyte subsets	Total Lymphocytes 855 (low) CD4 282 (low) CD8 299 (normal) CD3 589 (low) B Lymphocytes 133 (normal) Natural Killer Lymphocytes 144 (normal)	Immune system dysfunction
Cytogenetics of BM Sample	Normal Karyotype	Haematological malignancy, including MDS
Next Generation Sequencing of BM Sample	No additional myeloid mutations detected	Haematological malignancy (both myeloid and lymphoid)
Congo red stain	Negative	Amyloid
BM Immunohistochemistry	CD71 erythroid precursors	Present on initial BM sample. These are usually absent in PRCA.
Telomere Length Analysis	Normal	Bone marrow failure syndromes

ANA = Anti-nuclear antibody; BM: Bone Marrow; CCP = Cyclic Citrullinated Peptide; CTD = Connective Tissue Disease; dsDNA = double stranded DNA; ENA = Extractable nuclear antigen; Epo = Erythropoietin; HIV = Human Immunodeficiency Virus; LPD: Lymphoproliferative disorder; MDS: Myelodysplastic Syndrome; NR = Normal Range; PET/CT = Positron Emission Tomography/Computed Tomography; PNH = Paroxysmal Nocturnal Haemoglobinuria; PRCA = Pure Red Cell Aplasia; RF = Rheumatoid Factor; SLE: Systemic Lupus Erythematosus; T-LGL: T-cell large granular lymphocytic leukaemia

**Figure 1:**
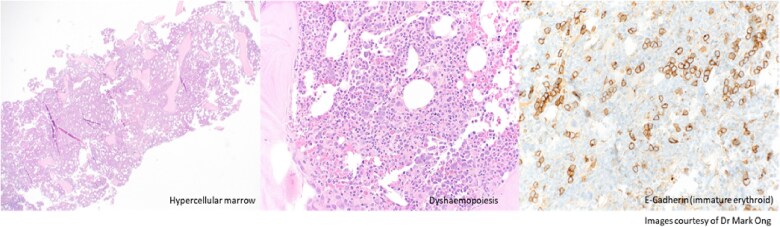
Bone marrow trephine biopsy (H&E and CD71 immunohistochemistry) showing hypercellular marrow with dyshaemopoiesis and scattered immature erythroid precursors (original magnification ×400). (courtesy of Dr Mark Ong, St Thomas’ histopathology department).

Bone marrow histology demonstrated hypercellular marrow with architectural disorder and some atypical megakaryocytes, as well as dyshaemopoiesis with no maturation arrest (Fig. 1). On review in our multidisciplinary team (MDT) meeting, overall consensus was that features were not typical for PRCA, especially with a normal reticulocyte count, and that the anaemia could be secondary to Filgotinib. Thus, initial recommendation was to continue holding this treatment and deliver supportive care with red cell transfusions.

As expected, after holding Filgotinib, the patient had severe flares from her rheumatoid arthritis and was treated with Rituximab and Prednisolone at 9 months after her initial presentation. Despite this, she continued to have persistent, refractory transfusion-dependent anaemia (Table 2).

**Table 2:** Summary of interventions with haemoglobin and reticulocyte levels during the patient’s treatment journey.

Date	Intervention	Haemoglobin (g/L)	Reticulocyte Count (×10*9/L)
14.12.2022	Filgotinib 200 mg OD	118	NA
29.12.2022	Filgotinib 200 mg OD	88	NA
6.2.2023	Filgotinib 200 mg OD on hold for 4 weeks	82	NA
15.2.2023	No treatment for RA	79	60
16.2.2023	Transfused 2 units PRBC	86	NA
30.3.2023	No treatment	78	81
11.6.2023	Transfused 2 units PRBC	59	NA
21.6.2023	Rituximab 1 g	84	NA
21.6.2023	Prednisolone 15 mg OD	84	NA
10.7.2023	Rituximab 1 g	107	44
13.9.2023	Prednisolone 15 mg OD now stopped	102	NA
20.10.2023	Transfused 3 units PRBC	69	NA
13.11.2023	Transfused 1 unit PRBC	77	NA
28.11.2023	Transfused 3 units PRBC	55	NA
7.12.2023	Erythropoietin (Neoreocormon; Epoetin Beta) 10 000 units once weekly s.c.	78	8
10.1.2024	Erythropoietin 10 000 units once weekly s.c.	81	NA
1.2.2024	Prednisolone 30 mg OD and Ciclosporin 50 mg BD. Erythropoietin continues.	101	174
15.2.2024	Prednisolone 15 mg OD and Ciclosporin 75 mg BD. Erythropoietin reduced to alternate weeks.	124	NA
22.2.2024	Prednisolone 10 mg OD and Ciclosporin 75 mg BD	134	44
1.3.2024	Prednisolone 5 mg OD and Ciclosporin 75 mg BD. Erythropoietin is stopped.	137	61
20.3.2024	Prednisolone stopped and Ciclosporin 75 mg BD	140	42
6.3.2025	Ciclosporin 75 mg BD	125	69
21.1.2026	Ciclosporin 75 mg BD	131	76

BD = Twice daily; NA = Not Available; OD = Once Daily; PRBC = Packed red blood cells; S.C. = Subcutaneously

Twelve months after initial presentation, the patient was anaemic with a new reticulocytopenia (Hb 78 g/l, Reticulocyte count 8 × 10*9/l). PRCA was considered as a potential diagnosis and, following MDT discussion, recommendation was to start Prednisolone 30 mg once daily with Ciclosporin A 50 mg twice a day.

This combination therapy was delayed due to the patient being hospitalised. However, once started, during the first month the patient managed to wean down Prednisolone by 5 mg every 7 days, and Erythropoietin injections were reduced to alternate weeks. She became transfusion-independent after four weeks and currently remains in complete remission on Ciclosporin A monotherapy (Table 2).

## Discussion

Acquired PRCA is characterised by severe anaemia and reticulocytopenia, with a broad differential including autoimmune disease, lymphoproliferative disorders, infections and medications (Table 3) [2]. PRCA secondary to RA is rare, with only limited case reports and variable clinical presentations described [3]. Diagnosis in RA patients may be challenging due to multifactorial causes of anaemia and the potential haematological toxicities associated with DMARD therapy [4].

**Table 3:** Classification of pure red cell aplasia.

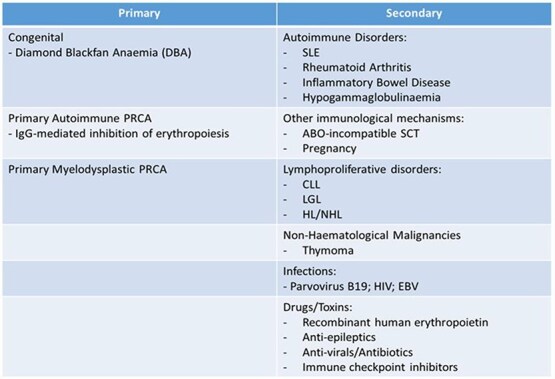

CLL: Chronic lymphocytic leukaemia; EBV: Epstein Barr virus; HIV: Human immunodeficiency virus; HL: Hodgkin lymphoma; LGL: Large granulocytic leukaemia; NHL: Non Hodgkin lymphoma; SCT: Stem cell transplant; SLE: Systemic Lupus Erythematosus [3]

One plausible explanation is that our patient had evolving RA-associated PRCA from the outset, which may initially have been obscured during Filgotinib treatment. Clinical trial data have demonstrated preserved or increased haemoglobin levels during the first months of Filgotinib therapy [5, 6]. Filgotinib selectively inhibits JAK1, whereas JAK2 is more closely associated with erythropoiesis and JAK3 with lymphocyte signalling [7]. It is therefore conceivable that preferential JAK1 inhibition may have transiently preserved erythropoietic signalling, contributing to the initially normal reticulocyte count before overt PRCA became apparent. This remains a hypothesis; larger pharmacovigilance studies are required.

Anti-erythropoietin antibody-mediated PRCA was also considered. However, the patient was already reticulocytopenic before commencing erythropoietin therapy, and the median reported onset of epoetin beta-associated PRCA is approximately 20 months [8].

Our patient did not respond to corticosteroids or Rituximab, both commonly used therapies for PRCA [2]. Increasing evidence suggests that PRCA associated with autoimmune disease may be predominantly T-cell mediated, which may explain the subsequent response to Ciclosporin A in this case [9]. Maintenance Ciclosporin A therapy has also been associated with sustained remission and improved outcomes [2, 10].

To our knowledge, this is the first reported case suggesting possible masking of PRCA during JAK1 inhibitor therapy in RA. Clinicians should consider PRCA in patients receiving JAK inhibitors who develop refractory anaemia, even in the presence of an initially normal reticulocyte count.

### Learning points

(i) JAK1 inhibitors may transiently normalise reticulocytes in occult PRCA
(ii) Persistent transfusion-dependent anaemia in RA requires serial reticulocyte monitoring
(iii) T-cell directed immunosuppression (Ciclosporin A) is effective when steroids and Rituximab fail
